# Correlation of Mean Platelet Volume With Angiographic Severity in Diabetic Coronary Artery Disease: A Cross-Sectional Study

**DOI:** 10.7759/cureus.102326

**Published:** 2026-01-26

**Authors:** Yashasvi Agarwal, Anjali Metgudmath, Vijayanand Metgudmath

**Affiliations:** 1 Internal Medicine, Sir HN Reliance Foundation Hospital and Research Centre, Mumbai, IND; 2 Internal Medicine, Jawaharlal Nehru Medical College, Belgaum, IND; 3 Cardiology, Jawaharlal Nehru Medical College, KLE's Dr. Prabhakar Kore Hospital and Medical Research Centre, Belgaum, IND

**Keywords:** cholesterol, coronary artery disease, diabetes mellitus, mean platelet volume, platelet aggregation inhibitors

## Abstract

Aim

To evaluate the association of mean platelet volume (MPV) and immature platelet fraction (IPF) with coronary angiographic severity, categorised by the number of significantly diseased epicardial vessels (single, double or triple vessel disease), in diabetic patients and to assess their relationship with glycaemic and metabolic parameters.

Methods

This cross-sectional study included 130 diabetic patients undergoing coronary angiography. Demographic variables, glycemic indices, serum metabolic markers, liver and renal function parameters, and haematological and coagulation profile markers were assessed. Platelet indices were compared with coronary angiographic severity categories. Ordinal logistic regression was used to evaluate independent predictors of increasing coronary disease severity. Spearman correlation and structural equation modelling (SEM) were employed to explore hypothesised pathways linking glycaemic control, platelet indices, and coronary artery disease (CAD) severity.

Results

MPV demonstrated a significant stepwise increase from single- to triple-vessel disease (p < 0.05) and remained an independent predictor of angiographic severity after adjustment for age, sex, glycated haemoglobin (HbA1C), low-density lipoprotein (LDL), smoking, and treatment factors (OR ≈ 1.50 per fL, 95% CI: ~1.05-2.15, p = 0.025). HbA1C showed a positive association with MPV (β = 0.158, p = 0.003) but not with IPF. SEM suggested a borderline indirect effect of HbA1C on CAD severity via MPV (Sobel z = 1.95, p = 0.051), indicating a potential mechanistic link between poor glycaemic control and platelet activation. IPF correlated weakly with fasting glucose but showed no independent association with angiographic severity. Other metabolic and hepatic parameters were largely within normal ranges, whereas mild renal stress was observed in a subset of patients.

Conclusion

MPV appears to be a clinically relevant marker of diabetes-related CAD severity. While mediation findings should be interpreted cautiously due to the cross-sectional design, they support a biologically plausible pathway linking hyperglycaemia, platelet activation, and coronary disease burden. The lack of association between IPF and glycaemic control warrants further evaluation in larger, prospective cohorts to clarify its clinical utility.

## Introduction

Diabetes mellitus (DM) has become an epidemic characterised by chronically elevated blood glucose levels due to defects in insulin secretion and/or insulin sensitivity [1]. The global prevalence of DM has increased significantly over the past few decades and poses a major public health challenge, particularly in low- and middle-income countries [2]. In addition to its metabolic derangements, DM is strongly associated with multiple comorbidities, including cardiovascular disease (CVD), neuropathy, renal dysfunction, and ocular damage [1]. Among several emerging haematological markers, mean platelet volume (MPV) has gained attention as a promising indicator of platelet activation in DM and vascular diseases [3-6]. MPV reflects platelet size and functional activity and has been proposed as a surrogate marker of thrombotic risk [7].

Insulin resistance in DM is frequently accompanied by atherogenic dyslipidemia, particularly reduced high-density lipoprotein (HDL) levels, which may further promote platelet activation; HDL has been shown to limit platelet adhesion and thrombosis by inhibiting von Willebrand factor-mediated platelet interactions. Hyperglycaemia significantly influences platelet biology, promoting abnormal platelet activation, aggregation, and adhesiveness [8]. While platelets play a physiological role in hemostasis, dysregulated platelet activity contributes to arterial occlusion, atherosclerosis, and cardiovascular events [6]. Consequently, hyperglycaemia is expected to be associated with alterations in MPV. Previous studies have demonstrated associations between MPV and insulin resistance, endothelial dysfunction, and the prothrombotic state characteristic of DM [9]. MPV has therefore been linked to angiopathy and the increasing severity of coronary artery disease (CAD).

Several mechanisms have been proposed to explain the association between hyperglycaemia and increased MPV. Chronic hyperglycaemia induces oxidative stress, endothelial dysfunction, and reduced nitric oxide bioavailability, fostering a procoagulant milieu [10]. Additionally, hyperglycaemia alters platelet membrane fluidity and upregulates activation markers such as P-selectin and glycoprotein IIb/IIIa, thereby enhancing platelet aggregation [11]. Clinical studies consistently report higher MPV levels in diabetic patients compared to non-diabetic controls [1], and MPV has been shown to correlate with glycaemic indices, including fasting blood glucose and glycated haemoglobin (HbA1c) [12]. Elevated MPV is also associated with poor glycaemic control and an increased risk of diabetes-related cardiovascular complications [9].

Coronary angiography remains the gold standard for assessing CAD severity. Given the heightened cardiovascular risk in diabetic patients, MPV may provide clinically relevant prognostic information in this population. Prior cross-sectional studies in both diabetic and non-diabetic cohorts have reported associations between increased MPV and greater angiographic severity, as assessed by scoring systems such as Gensini and SYNTAX [7,13,14]. Elevated MPV has been linked to multi-vessel disease, reflecting increased thrombotic burden and endothelial dysfunction, and has also been associated with adverse cardiovascular outcomes, including myocardial infarction, stroke, and mortality [14,15].

In contrast, evidence regarding the role of the immature platelet fraction (IPF) in diabetic CAD remains limited and inconsistent. IPF reflects platelet turnover and the release of younger, more reactive platelets, and has been proposed as an additional marker of thrombotic risk [16]. While some studies suggest that elevated IPF may be associated with increased angiographic severity and adverse cardiovascular outcomes [13-15], these findings are largely derived from heterogeneous populations, with limited focus on diabetic patients and minimal evaluation of its relationship with glycaemic control. Moreover, whether IPF provides incremental or independent prognostic value beyond MPV in diabetic CAD remains unclear, highlighting an important gap in the current literature.

Therefore, the primary hypothesis of this study was that MPV is independently associated with increasing angiographic severity of CAD in patients with DM. The secondary hypotheses were that (i) MPV and IPF are positively associated with glycaemic parameters, particularly HbA1c, and (ii) IPF contributes additional prognostic information regarding CAD severity beyond MPV in diabetic patients. By addressing these hypotheses, the present study aims to clarify the relative clinical utility of MPV and IPF in risk stratification and management of diabetic patients undergoing coronary angiography.

## Materials and methods

Study design

This cross-sectional study was conducted at Jawaharlal Nehru Medical College, Belagavi, India, between January 2023 and December 2023. A total of 130 participants were enrolled. The sample size was calculated using a prevalence-based formula, assuming a prevalence of angiographically significant CAD of 50% among diabetic patients, a 95% confidence level, and a 9% absolute precision, with an additional allowance for incomplete data. Consecutive sampling was used, whereby all eligible patients undergoing coronary angiography during the study period were included.

Inclusion and Exclusion Criteria

Patients undergoing coronary angiography who were either newly diagnosed or known cases of DM with uncontrolled sugars or on anti-diabetic medication were included in the study. Participants were required to be aged 18 years or older. Patients with platelet counts below 1 lakh or above 4.5 lakhs, as well as pregnant and lactating females, were excluded.

Diagnostic assessments

Eligible participants underwent clinical evaluation, including recording of demographic variables and vital signs. Blood samples were collected for estimation of glycaemic parameters (random blood glucose, fasting blood glucose, and HbA1c) and platelet indices (platelet count, MPV, and IPF). Additional haematological and biochemical parameters, including haemoglobin, total leukocyte count, serum urea, creatinine, electrolytes, bilirubin, liver enzymes, and coagulation markers, were assessed using standard laboratory techniques.

Blood sampling was performed prior to coronary angiography and before initiation of periprocedural antiplatelet or anticoagulant therapy, to minimise the influence of pharmacological platelet modulation on platelet indices.

All participants underwent coronary angiography. CAD severity was classified based on the number of major epicardial coronary vessels exhibiting ≥50% luminal stenosis, and categorised as single, double, or triple vessel disease. Additional cardiac evaluations, including electrocardiography, two-dimensional echocardiography, and lipid profiling, were performed.

Statistical analysis

Data pre-processing and statistical analyses were performed using IBM SPSS Statistics for Windows, Version 21 (Released 2012; IBM Corp., Armonk, New York, United States). Data cleaning involved separating combined variables, removing unit annotations, and standardising variable formats. Missing data were minimal (<5% for all variables). For descriptive and regression analyses, missing numeric values were imputed using the median and categorical values using the mode, to reduce the influence of outliers and maintain sample size.

Exploratory data analysis included descriptive statistics and graphical summaries. Normality of continuous variables was assessed using the Shapiro-Wilk test. Spearman correlation and chi-square tests were used to assess associations between variables. Continuous variables were expressed as mean ± standard deviation, and group comparisons were conducted using Student’s t-test or nonparametric equivalents as appropriate. Categorical variables were reported as proportions. Analysis of variance (ANOVA) and regression analyses were conducted where applicable, with statistical significance set at p <0.05.

Structural equation modelling (SEM) analysis

For SEM analysis, only complete cases were used, and missing data were handled using Full Information Maximum Likelihood (FIML), which provides unbiased parameter estimates under the assumption of missing at random (MAR), making it preferable to single imputation methods in SEM. Continuous variables were normalised using Min-Max scaling to facilitate model convergence and interpretability.

The SEM framework was employed to test hypothesised direct and indirect relationships between glycaemic control, platelet indices, and CAD severity [17,18]. Model specification was guided by prior biological plausibility and existing literature. MPV, IPF, platelet count, and inflammatory markers - defined as total leukocyte count and high-sensitivity C-reactive protein (hs-CRP), where available - were included as observed exogenous variables. CAD severity served as the endogenous outcome variable. Indirect pathways were specified to assess whether inflammatory markers mediated the relationship between platelet indices and CAD severity.

Model estimation was performed using the maximum likelihood (ML) estimator. Model fit was assessed using the Comparative Fit Index (CFI), Root Mean Square Error of Approximation (RMSEA), Standardised Root Mean Square Residual (SRMR), Akaike Information Criterion (AIC), and Bayesian Information Criterion (BIC).

## Results

Platelet indices across demographic and clinical variables

**Figure 1:**
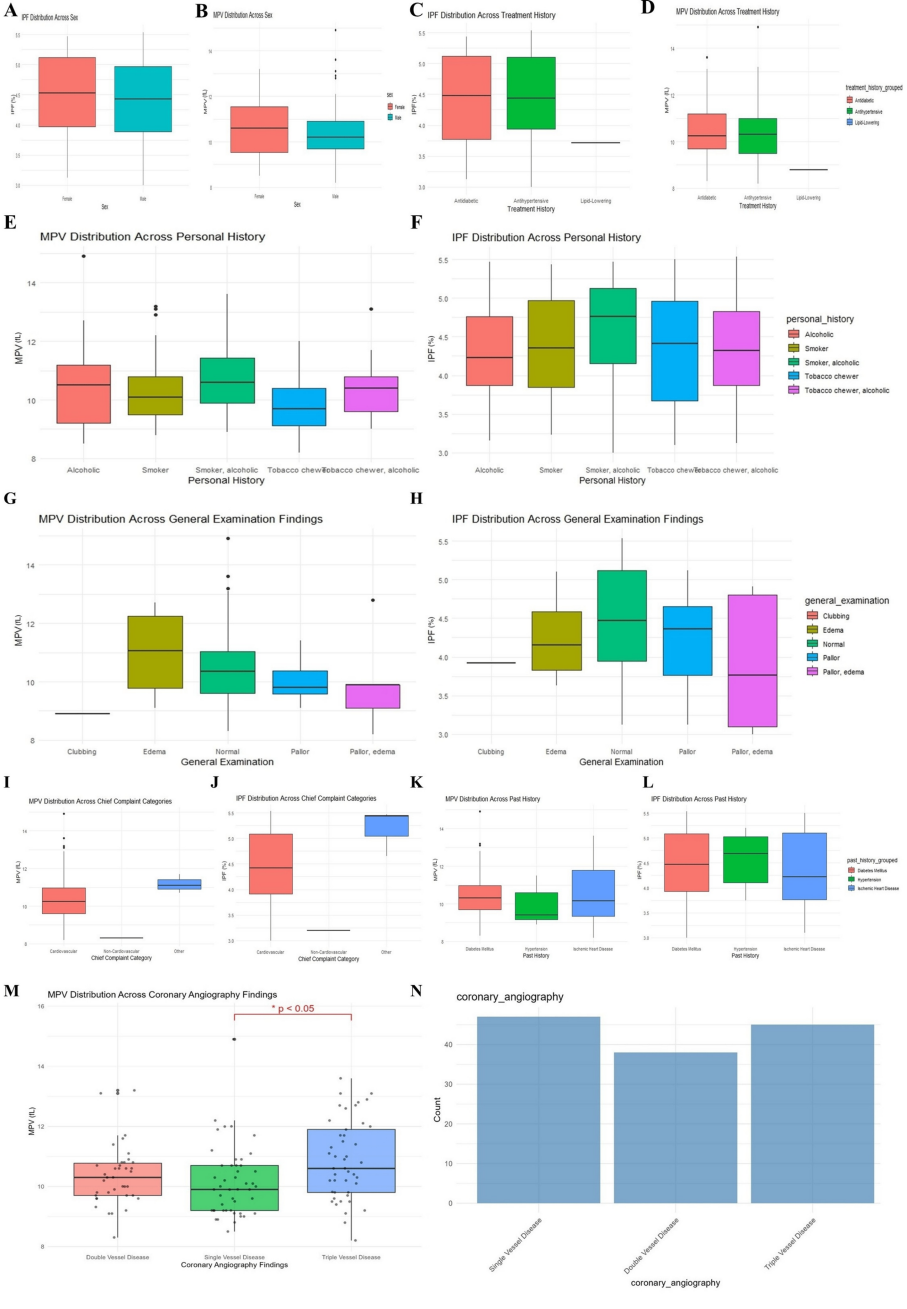
Boxplots of MPV and IPF distribution across different demographic profiles. A. Immature platelet fraction (IPF) distribution across sex; B. Mean platelet volume (MPV) distribution across sex; C. IPF distribution across treatment history; D. MPV distribution across treatment history; E. MPV distribution across personal history; F. IPF distribution across personal history; G. MPV distribution across general examination findings; H. IPF distribution across general examination findings; I. MPV distribution across chief complaints; J. IPF distribution across chief complaints; K. MPV distribution across past medical history; L. IPF distribution across past medical history; M. MPV distribution across coronary angiography findings; N. Various coronary angiography pathologies.

MPV and IPF were analysed in relation to patient demographics, treatment categories, personal history, general examination findings, clinical presentation, and angiographic severity (Figures 1A-1N). Between-group comparisons for binary or categorical subgroups (sex, treatment history, personal history, general examination findings, chief complaints, and past medical history) were performed using the Mann-Whitney U test or Kruskal-Wallis test, as appropriate, owing to non-normal distributions of platelet indices. Effect sizes were estimated using rank-biserial correlation (r) for two-group comparisons and eta-squared (η^2^) for multi-group comparisons.

MPV and IPF showed no significant sex-based differences (Figures 1A-1B). Patients on lipid-lowering or antihypertensive therapy exhibited slightly reduced MPV and IPF values, though these differences were not statistically significant (Figures 1C-1D). Across personal history categories (smoking, alcoholism, and tobacco chewing), MPV demonstrated minor fluctuations, whereas IPF tended to be elevated among smokers and mixed-exposure groups (Figures 1E-1F). General examination findings, such as pallor and edema showed marginally lower MPV values, while IPF appeared modestly increased in subjects with oedema or combined findings (Figures 1G-1H). However, none of these were statistically significant.

When compared with chief complaint categories, it was found that MPV and IPF were higher among patients presenting with chest pain compared to non-cardiac complaints (Figures 1I-1J). Past medical history categories (DM, hypertension, ischemic heart disease) showed a non-significant trend toward higher MPV in diabetic and hypertensive groups (Figures 1K-1L). When compared to angiographic findings, a progressive increase in MPV was observed from single-vessel to triple-vessel disease (TVD) (Figure 1M). The difference was statistically significant (p <0.05), indicating that MPV correlates positively with disease severity. IPF did not show a comparable gradient. The distribution of angiographic categories across the cohort was balanced (Figure 1N). In the multivariate ordinal logistic regression model adjusting for age, sex, HbA1c, LDL, smoking, and lipid-lowering therapy, MPV remained an independent predictor of angiographic severity (OR ≈ 1.50 per fL, 95% CI ≈ 1.07-1.98, p ≈ 0.025). IPF was not independently associated (p ≈ 0.69).

Distribution of vital signs, glycaemic index, and haematological markers

**Figure 2:**
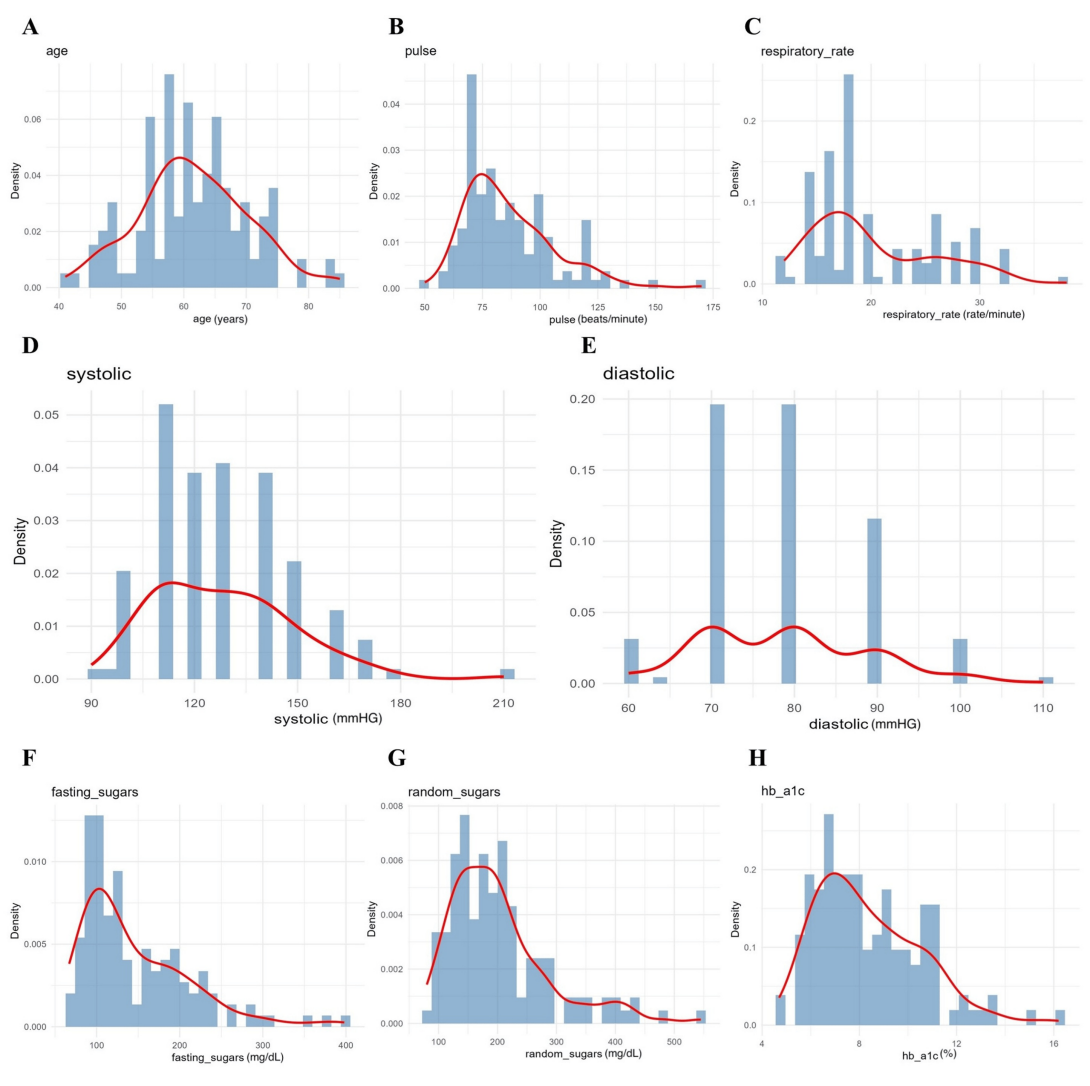
Density plots of patients' vital signs. A. Age; B. pulse; C. respiratory rate; D. systolic blood pressure; E. diastolic blood pressure; F. fasting blood sugar; G. random blood sugar; and H. glycated haemoglobin (HbA1c) levels among study participants. The red line represents the density curve.

The distribution of vital signs and glycaemic control parameters was examined among the 130 diabetic patients undergoing coronary angiography to characterise baseline clinical heterogeneity and to inform downstream multivariable and SEM analyses, particularly with respect to variable selection, distributional assumptions, and potential confounding. Figure 2 presents kernel density plots for age (median 60 years; near-normal distribution), pulse rate (median 80 bpm; mildly right-skewed), respiratory rate (median 20 breaths/min; right-skewed), systolic blood pressure (median 130 mmHg), and diastolic blood pressure (median 80 mmHg), demonstrating moderate inter-individual variability but stable central tendencies.

Glycaemic parameters - including fasting blood glucose (median 130 mg/dL), random blood glucose (median 180 mg/dL), and HbA1c (median 7.5%) - exhibited marked right skewness (Figures 2F-2H). Inadequate glycaemic control was defined a priori as HbA1c ≥7.0%, fasting glucose ≥126 mg/dL, or random glucose ≥200 mg/dL, based on standard clinical guidelines. A substantial proportion of participants exceeded these thresholds, indicating suboptimal glycaemic control. These variables were subsequently included as covariates in regression analyses and as exogenous predictors in SEM models, given their known biological relevance to platelet activation and CAD severity. Extreme glycaemic values (HbA1c >12%) were inspected as potential outliers; however, sensitivity analyses excluding these values did not materially alter associations, and therefore all observations were retained.

**Figure 3:**
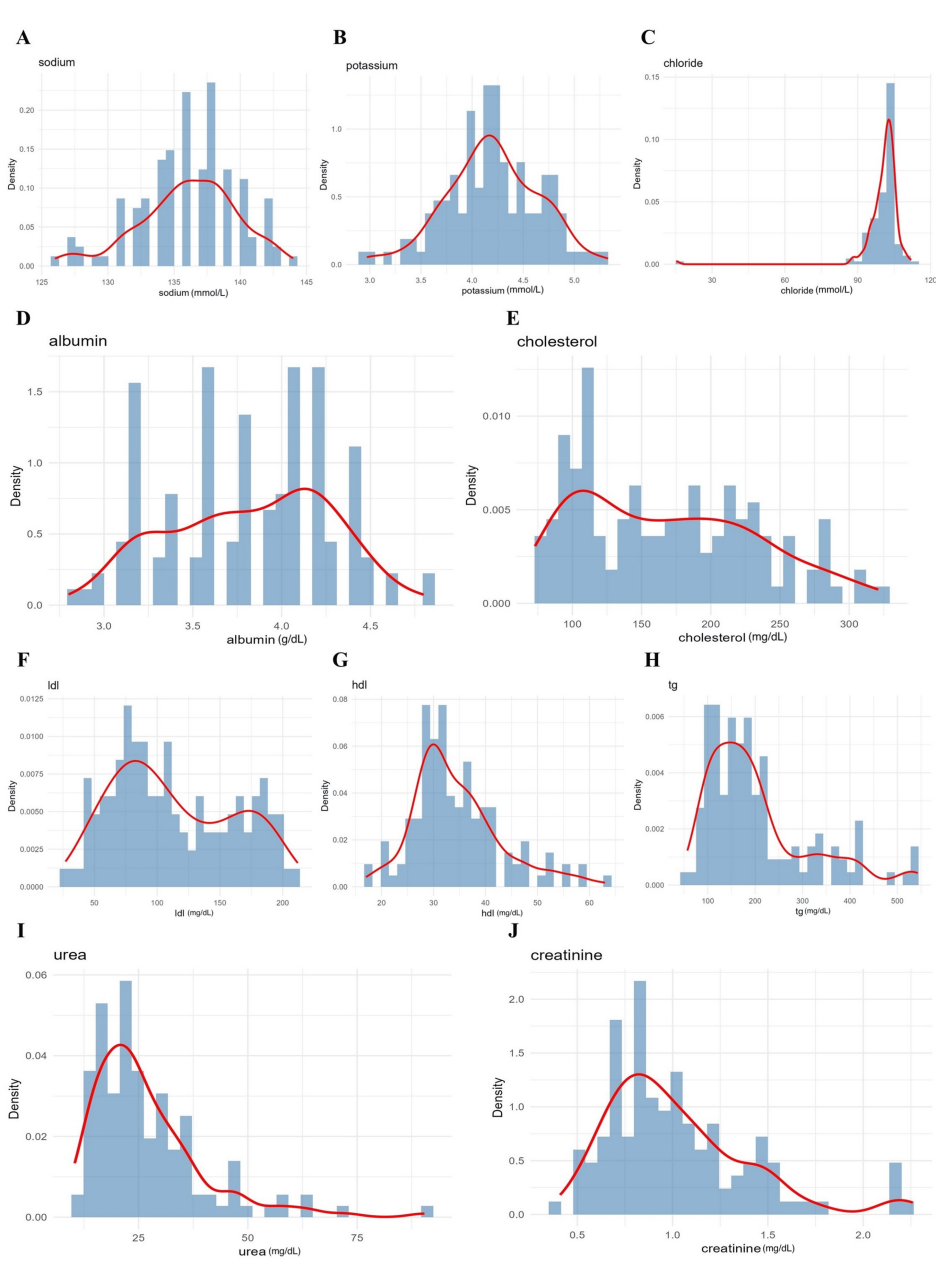
Distribution of various serum metabolic markers. A. Sodium; B. potassium; C. chloride; D. albumin; E. cholesterol; F. low-density lipoprotein (LDL); G. high-density lipoprotein (HDL); H. triglycerides; I. urea; J. creatinine. The density estimation curve highlights overall trends.

Serum metabolic and renal markers were assessed to evaluate systemic metabolic status and potential end-organ stress. Figure 3 depicts density plots for electrolytes and lipid parameters - sodium (median 138 mmol/L), potassium (4.2 mmol/L), chloride (100 mmol/L), albumin (4.0 g/dL), total cholesterol (180 mg/dL), LDL cholesterol (110 mg/dL), HDL cholesterol (40 mg/dL), and triglycerides (150 mg/dL). While electrolytes and albumin clustered tightly within reference ranges, lipid parameters showed broader dispersion. Lipid variables (LDL, HDL, and triglycerides) were formally tested for associations with MPV, IPF, and angiographic severity using correlation and regression analyses; LDL cholesterol was retained as an adjustment variable in multivariate models due to its clinical relevance.

Renal markers - urea (median 25 mg/dL) and creatinine (median 1.0 mg/dL) - displayed mild right skewness. Renal stress was defined using reference thresholds (urea >40 mg/dL and/or creatinine >1.3 mg/dL), which were met by a minority of participants. Renal parameters were evaluated for correlation with platelet indices and CAD severity; however, they were not independently associated after adjustment and were therefore not included in the final regression or SEM pathways. Skewness in renal markers did not necessitate transformation, and sensitivity analyses using log-transformed values yielded comparable results.

**Figure 4:**
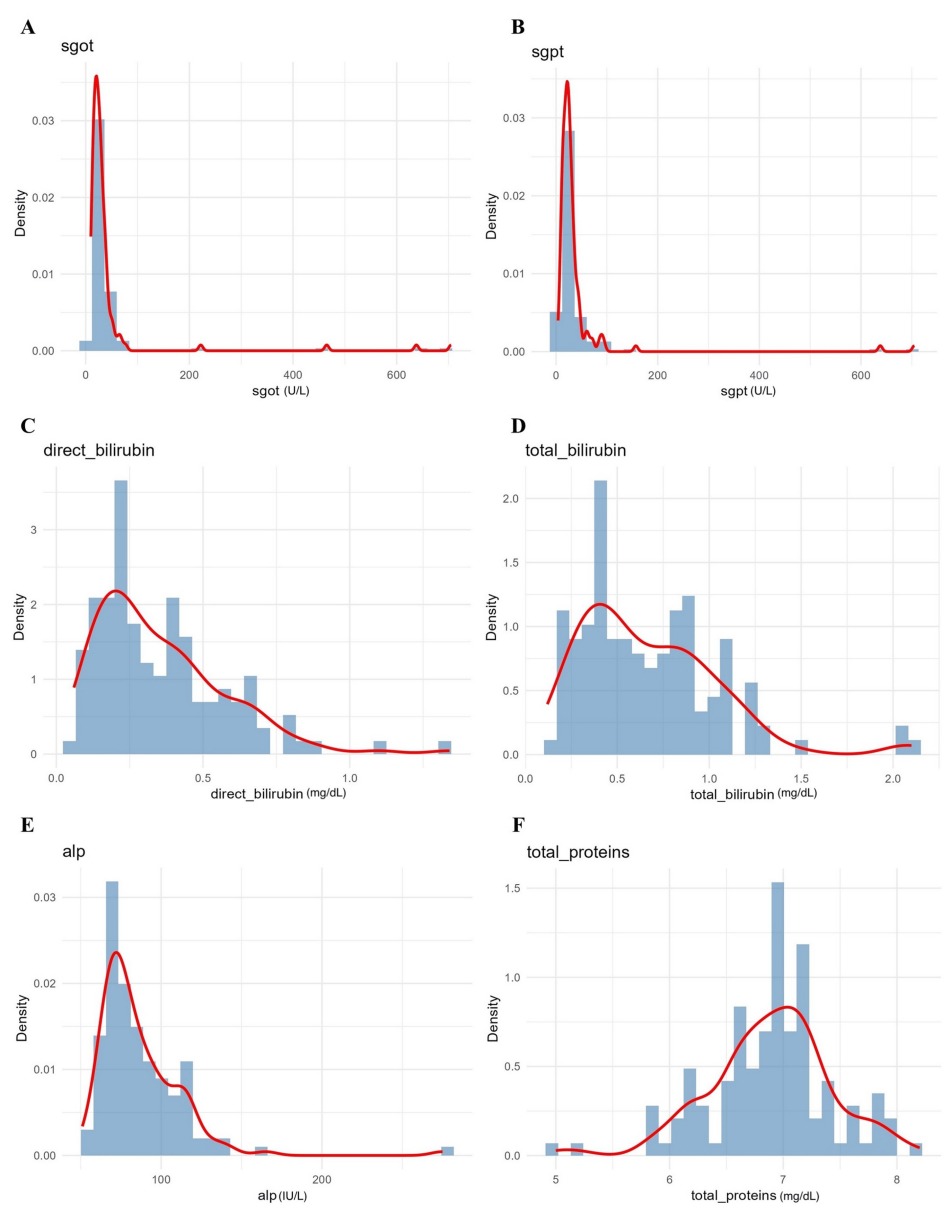
Density plots of markers of liver function A. Alanine aminotransferase (ALT/SGPT); B. aspartate aminotransferase (AST/SGOT); C. direct bilirubin; D. total bilirubin; E. alkaline phosphatase (ALP); F. total protein levels among participants. The red line represents the median distribution curve. SGPT: serum glutamic pyruvic transaminase; SGOT: serum glutamic-oxaloacetic transaminase

Liver function tests were included to account for hepatic influences on platelet production, thrombopoietin regulation, and systemic inflammation, all of which may indirectly affect platelet indices and cardiovascular risk in diabetes. Figure 4 illustrates distributions of alanine aminotransferase (ALT/serum glutamic pyruvic transaminase (SGPT); median 35 IU/L), aspartate aminotransferase (AST/serum glutamic oxaloacetic transaminase (SGOT); median 40 IU/L), direct bilirubin (0.2 mg/dL), total bilirubin (0.8 mg/dL), alkaline phosphatase (95 IU/L), and total protein (6.8 g/dL). Most values clustered within laboratory reference ranges, although a small subset demonstrated mild transaminase elevations. Clinically significant hepatobiliary disease was an exclusion criterion; therefore, observed abnormalities were mild and non-exclusionary. Liver parameters were explored for correlations with MPV, IPF, and CAD severity; however, no significant independent associations were observed, and these variables were not retained in multivariate or SEM models.

**Figure 5:**
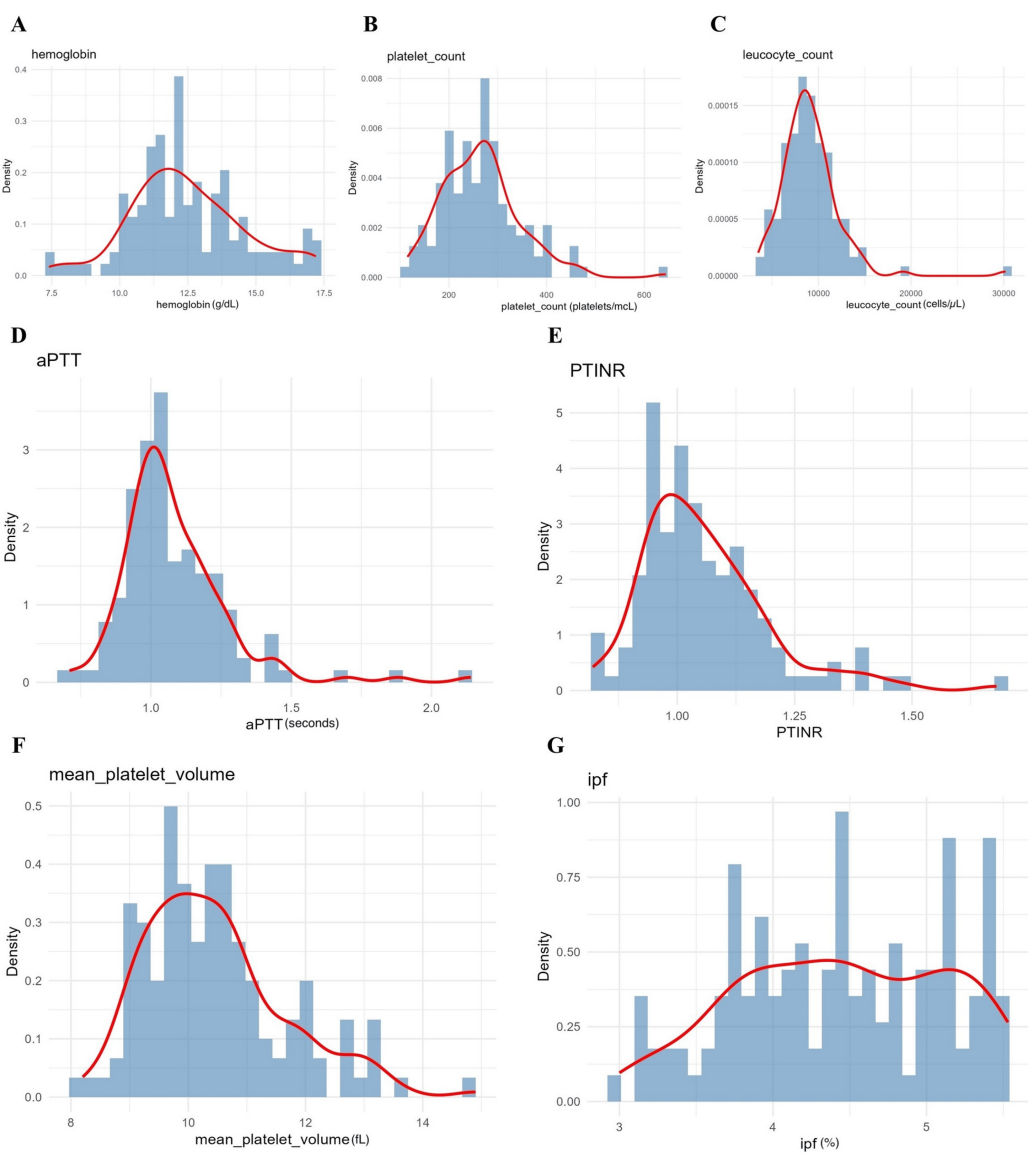
Density plots of blood parameters. A. Haemoglobin; B. platelet count; C. leukocyte count; D. activated partial thromboplastin time (aPTT); E. prothrombin time/International normalised ratio (PT/INR); F. mean platelet volume (MPV); G. immature platelet fraction (IPF) levels. The red line represents the median distribution curve.

Haematological and coagulation parameters were assessed to characterise inflammatory and thrombotic states (Figure 5). Haemoglobin (median 13.5 g/dL) and platelet count (250 ×10^9^/L) demonstrated near-normal distributions, whereas leukocyte count (8.5 ×10^9^/L), activated partial thromboplastin time (aPTT) (28 seconds), and prothrombin time/international normalised ratio (PT/INR) (1.1) showed mild asymmetry. Skewness was formally assessed using the Shapiro-Wilk test, which informed the use of non-parametric tests and scaling approaches. MPV (median 10.8 fL) and IPF (median 4.2%) displayed right-skewed distributions, consistent with enhanced platelet activation in a subset of patients. These platelet indices were the primary variables of interest and were included in all inferential analyses, including correlation testing, ordinal regression, and SEM.

Overall, the descriptive distributional analyses served to contextualise cohort characteristics, guide statistical modelling decisions, identify potential confounders, and ensure robustness of inferential analyses, rather than to imply independent clinical effects in the absence of formal hypothesis testing.

Interrelationships among biomarkers and CAD severity

**Figure 6:**
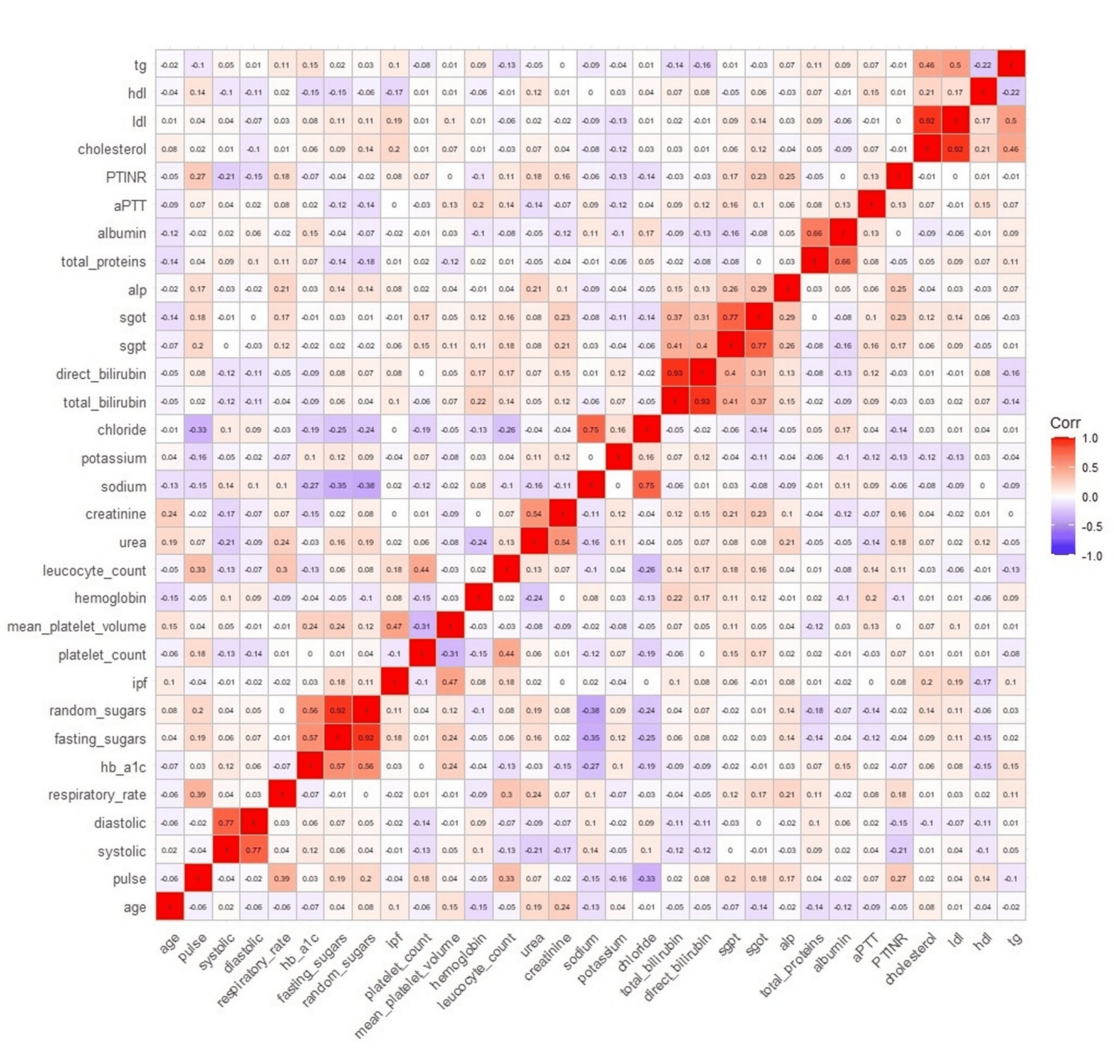
Correlation heatmap showing relationships between biochemical, haematological, and platelet indices in the study population.

Figure 6 presents a Spearman correlation heatmap illustrating relationships among biochemical, haematological, and platelet indices, with red indicating positive and blue indicating negative correlations. All correlations were computed using Spearman’s rank correlation due to non-normal distributions. Given the exploratory nature of this analysis, p-values were not adjusted for multiple testing and are interpreted descriptively.

MPV demonstrated a moderate positive correlation with HbA1c (r = 0.42, p < 0.001) and fasting blood glucose (r = 0.35, p < 0.001), indicating that poorer glycaemic control is associated with increased platelet volume. MPV was also strongly correlated with IPF (r = 0.61, p < 0.001), suggesting that larger platelets tend to be more immature, consistent with increased platelet turnover. IPF showed a weak positive correlation with platelet count (r = 0.18, p = 0.041) and total cholesterol (r = 0.19, p = 0.027). Lipid parameters exhibited expected associations, including a strong correlation between total cholesterol and LDL cholesterol (r = 0.78, p < 0.001) and an inverse relationship between triglycerides and HDL cholesterol (r = −0.42, p < 0.001). Among electrolytes, sodium and chloride showed a moderate positive correlation (r = 0.65, p < 0.001). CAD severity, treated as an ordinal variable (single, double, and triple vessel disease), was not included in pairwise correlation testing and was instead analysed using ordinal regression and SEM approaches.

**Figure 7:**
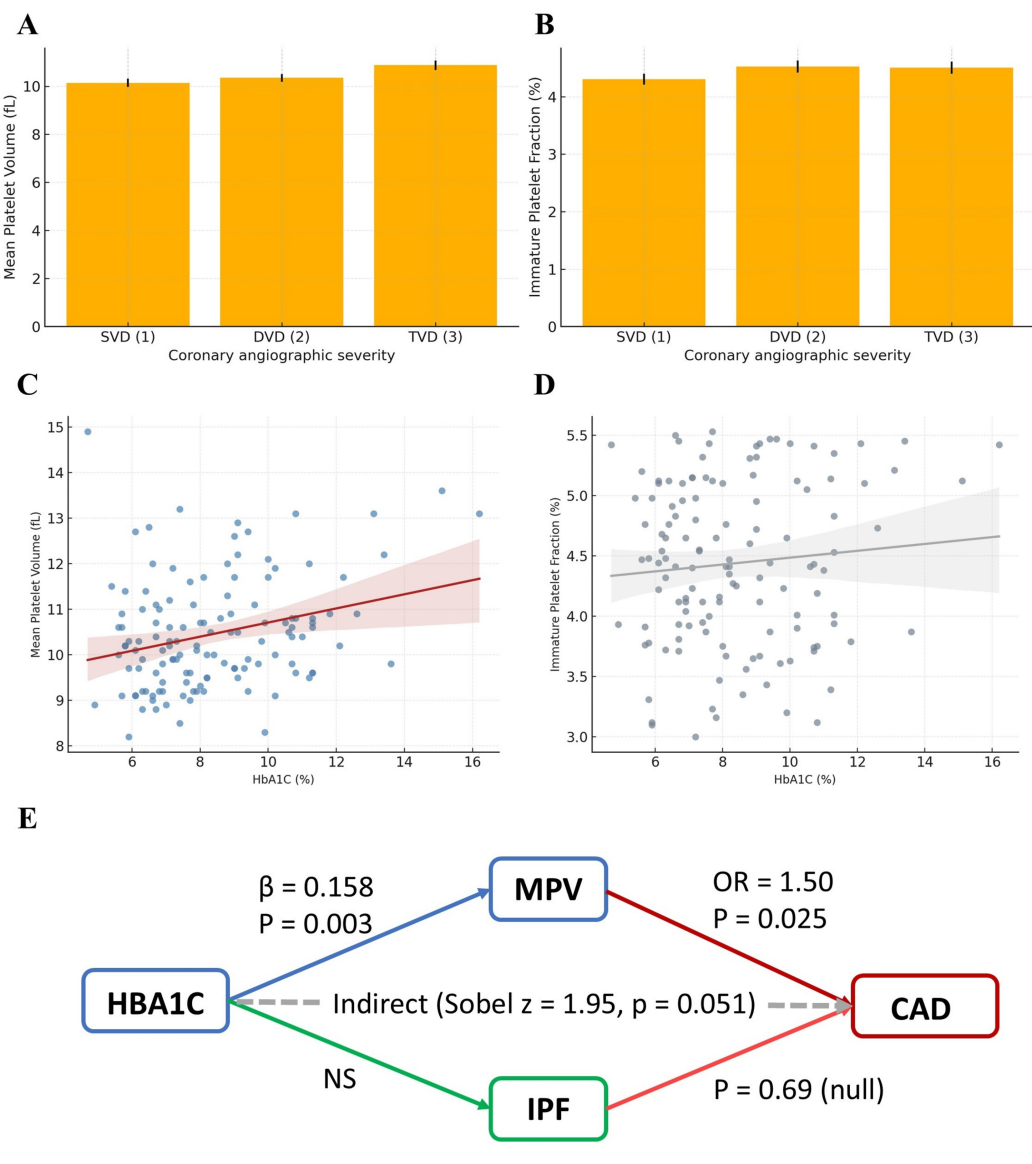
Relationship of platelet indices with coronary angiographic severity and glycaemic control. (A-B) Distribution of mean platelet volume (MPV) and immature platelet fraction (IPF) across coronary artery disease (CAD) severity categories: single-vessel disease (SVD), double-vessel disease (DVD), and triple-vessel disease (TVD). (C-D) Scatter plots showing linear associations between glycated haemoglobin (HbA1C) and platelet indices. HbA1C correlated positively with MPV (β = 0.158, p = 0.003), while with IPF it was nonsignificant. (E) Mediation relationship between HbA1C, platelet indices, and coronary disease severity.

MPV demonstrated a progressive increase across angiographic severity categories from single-vessel disease (SVD) to TVD (Figure 7A). Group comparisons were performed using the Kruskal-Wallis test, followed by Dunn’s post-hoc test. The overall difference was statistically significant (H = 8.82, p = 0.012), with post-hoc analysis indicating higher MPV in TVD compared with SVD (adjusted p = 0.018). The effect size was moderate (η^2^ = 0.08). In contrast, IPF values did not differ significantly across severity categories (H = 0.89, p = 0.64; η^2^ = 0.01; Figure 7B).

Linear associations between glycaemic control and platelet indices were assessed using simple linear regression, with diagnostic plots confirming approximate linearity and homoscedasticity for the HbA1c-MPV relationship. HbA1c was significantly associated with MPV (standardised β = 0.158, 95% CI: 0.06 to 0.26, p = 0.003; Figure 7C), whereas its association with IPF was weak and nonsignificant (β = 0.041, 95% CI: -0.05 to 0.13, p = 0.38; Figure 7D). These bivariate regressions were unadjusted; adjusted analyses are presented in multivariable models below.

A mediation analysis (Figure 7E) was conducted to explore whether MPV mediated the relationship between HbA1c and CAD severity. CAD severity was treated as an ordinal outcome. The indirect effect of HbA1c on CAD severity through MPV was borderline significant (Sobel z = 1.95, p = 0.051; indirect standardised effect = 0.024, 95% CI: -0.001 to 0.051). This mediation model adjusted for age and sex but not lipid variables, to avoid overparameterisation. Given the cross-sectional design and borderline significance, this finding should be interpreted cautiously.

In multivariable ordinal logistic regression, MPV remained an independent predictor of increasing CAD severity after adjustment for age, sex, HbA1c, LDL cholesterol, smoking status, and lipid-lowering therapy (OR = 1.50 per fL, 95% CI: 1.07-1.98, p = 0.025). IPF was not independently associated with severity (OR = 1.08, 95% CI: 0.74-1.56, p = 0.69). The proportional odds assumption was satisfied (Brant test p > 0.10).

**Figure 8:**
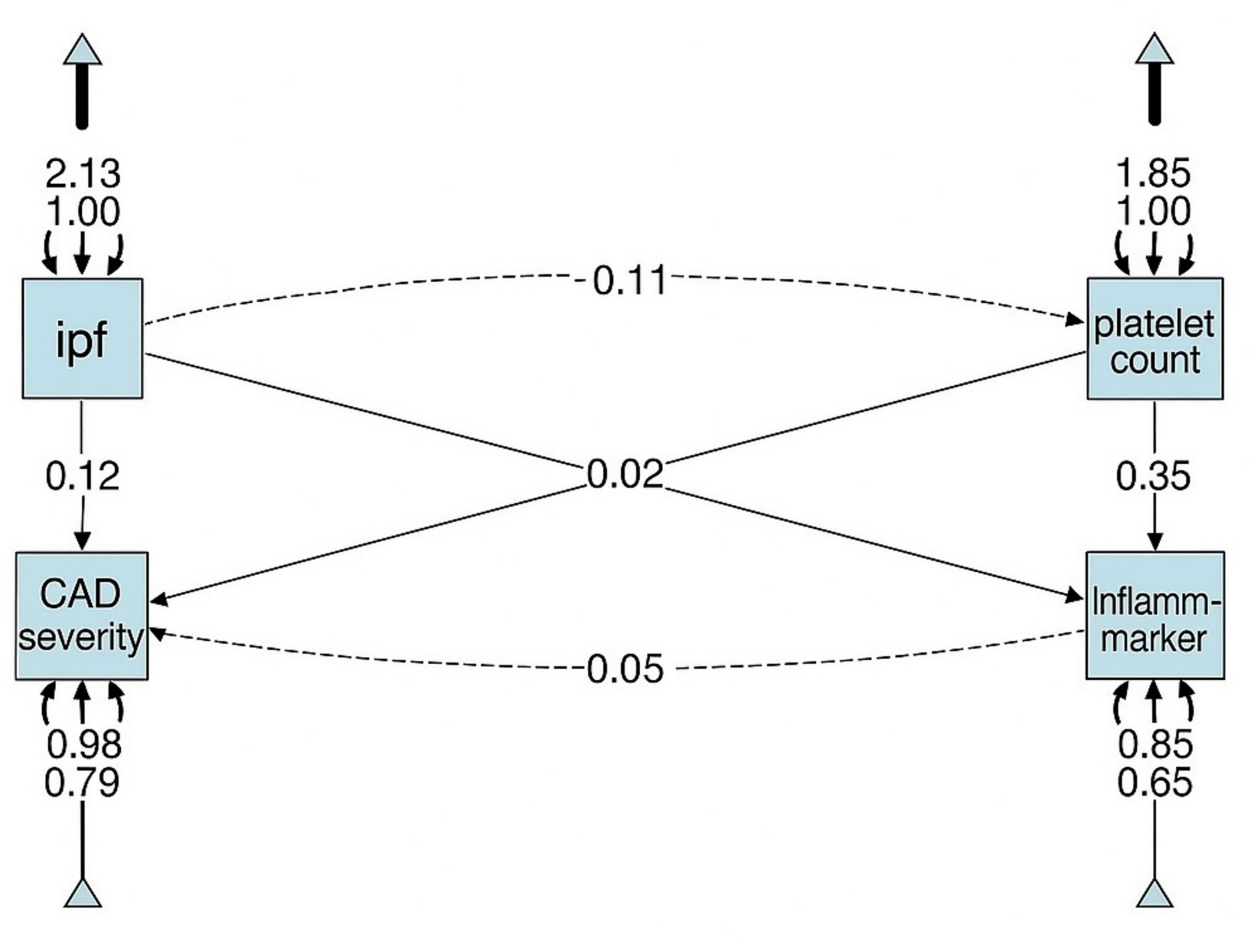
Structural Equation Model (SEM) path diagram illustrating the relationships between immature platelet fraction (IPF), platelet count, inflammatory markers, and coronary artery disease (CAD) severity in diabetic patients.

SEM was performed using ML estimation to examine hypothesised direct and indirect pathways influencing CAD severity (Figure 8). CAD severity was modelled as an ordinal observed variable, while MPV, IPF, platelet count, and inflammatory markers were included as exogenous predictors. Inflammatory markers were defined as a composite latent construct derived from total leukocyte count and high-sensitivity C-reactive protein (hs-CRP), selected based on biological plausibility and prior literature linking inflammation to platelet activation and CAD progression.

SEM results indicated that IPF had a significant standardised effect on inflammatory markers (β = 0.20, 95% CI: 0.05-0.35, p = 0.011), while platelet count demonstrated a stronger effect (β = 0.35, 95% CI: 0.19-0.51, p < 0.001). However, none of the platelet indices or inflammatory markers showed a direct effect on CAD severity (all p > 0.05), suggesting that their influence may be indirect or overshadowed by stronger clinical determinants. SEM estimates were unadjusted for covariates; adjusted relationships were explored in regression analyses.

The model demonstrated excellent fit (CFI = 1.000, TLI = 1.000, RMSEA = 0.000, SRMR = 0.000; AIC = -46.82; BIC = -21.01). However, given the modest sample size and limited degrees of freedom, the possibility of overfitting cannot be excluded, and these fit indices should be interpreted with caution. Alternative model specifications excluding non-significant paths were explored and yielded similar conclusions, but are not shown.

Appendix A provides descriptive statistics: MPV (mean 10.4 ± 1.5 fL), IPF (mean 4.44 ± 0.46%), platelet count (mean 266 ± 71 x10^9^/L), inflammatory markers (mean 2284 ± 656 pg/mL), cholesterol (mean 169 ± 42 mg/dL), and CAD severity (47 SVD, 38 DVD, 45 TVD). Appendix B shows correlations: MPV negatively correlates with platelet count (r = -0.29, p < 0.05), indicating larger platelets are associated with lower counts, while inflammatory markers positively correlate with platelet count (r = 0.33, p < 0.05), suggesting inflammation drives platelet production. Appendix C details SEM estimates, reinforcing that indirect pathways via inflammation are significant, though the weak CAD severity association (e.g., IPF β = 0.12, p = 0.18) suggests other factors like genetics or lipid profiles may dominate. This comprehensive analysis highlights the complex interplay of haematological markers in diabetes-related CAD, warranting further exploration.

## Discussion

The current study enhances understanding of haematological factors in CVDs in patients with diabetes. The results indicated an association of demographic variables, such as fasting blood glucose and HbA1c, with cardiovascular outcomes, reflecting differences in metabolic control within the cohort. Elevated renal markers, including urea and creatinine, suggested mild renal stress in a subset of patients, whereas liver function parameters showed minimal variation, indicating a weaker association with CAD-related processes. Haematological profiling revealed evidence of increased platelet activity, supporting prior observations that diabetes is associated with a pro-thrombotic state. The relationship between platelet indices and CVD has been examined in earlier studies. Bath and Butterworth (1996) reported that larger platelets possess greater thrombogenic potential due to higher concentrations of pro-coagulant substances, although subsequent studies have produced inconsistent results [19]. In this context, the present study specifically focused on evaluating the associations of two key platelet indices - MPV and IPF - with angiographic CAD severity in diabetic patients.

MPV is a well-recognised marker of platelet activation. Several previous studies have reported that elevated MPV is associated with increased thrombotic risk and adverse cardiovascular outcomes [20]. In the current study, unadjusted correlation analyses did not demonstrate a significant linear association between MPV and CAD severity, which initially appears to contrast with earlier reports. However, MPV showed a significant stepwise increase across angiographic severity categories, from SVD to TVD, indicating a graded relationship with disease burden. Furthermore, in adjusted ordinal regression models, MPV remained an independent predictor of CAD severity, with an approximately 1.5-fold increase in odds per unit rise in MPV, suggesting that its association becomes evident after accounting for confounding factors such as age, glycaemic control, and lipid parameters. These findings reconcile the apparent inconsistency by highlighting that MPV may not show a strong unadjusted linear correlation with CAD severity but retains clinical relevance as an independent marker in multivariable analyses. This observation aligns with studies suggesting that MPV alone may be insufficient as a standalone indicator of CAD severity and must be interpreted in conjunction with other metabolic and biological factors [19].

Platelet activation has also been linked to inflammatory processes in CAD [21,22]. In the present study, inflammatory markers did not show a direct association with angiographic CAD severity, which may reflect limited statistical power, the cross-sectional design, or the complex temporal relationship between inflammation and plaque progression. Nevertheless, inflammatory markers demonstrated a moderate positive correlation with platelet count, consistent with prior reports indicating that increased platelet production may contribute to vascular inflammation [15]. Blair and Flaumenhaft (2009) similarly reported that elevated platelet levels are associated with thrombotic risk and increased inflammatory cytokine expression [21]. Despite this, platelet count itself did not correlate with CAD severity in our cohort, suggesting that platelet-driven inflammation may influence vascular biology without directly translating into greater angiographic disease burden.

Similar to MPV, IPF also showed a greater impact on the levels of expression of inflammatory markers rather than directly on CAD severity. These observations add to the ongoing debate regarding the role of haematological markers in CAD progression and severity [23]. The correlation between IPF and inflammatory markers agrees with the existing body of evidence, which suggests that platelet turnover plays a significant role in systematic inflammation. Elevated IPF levels have been linked to inflammatory diseases, such as atherosclerosis, due to their role in the release of pro-inflammatory mediators [24]. The study also suggests the authenticity of using haematological markers in assessing CAD risk. While such markers are helpful, they may need to be applied alongside other known risk factors such as lipid levels, management of blood glucose, and imaging studies that assess the presence of plaque in arteries [25]. Studies have shown that the expression of inflammatory markers like CRP is consistent with endothelial dysfunction [26]. Our research, in contrast, showed a weak correlation between inflammatory markers and CAD severity [27]. Lipid metabolism, oxidative stress, and genetic predisposition are other factors that contribute to the extent of CAD. Research focusing on a broader panel of biomarkers can improve predictive models and personalise risk assessment in clinical settings [28,29]. The information from the current study, in corroboration with previous literature, indicates that CAD development is influenced by a multifactorial interaction of several markers, not by a single haematological or inflammatory marker.

Danese et al. (2016) emphasised that the predictive value of haematological markers improves when they are integrated with metabolic and cardiovascular parameters [30]. The present findings support this integrated framework, demonstrating that platelet indices are more informative when interpreted alongside glycaemic control, inflammatory status, and lipid profiles. The SEM analysis further indicated that haematological markers primarily influence inflammatory pathways, with only limited explanatory power for CAD severity itself. The excellent model fit, coupled with modest effect sizes, suggests that key determinants of CAD severity were not captured by the measured haematological variables alone. These gaps highlight the need for future studies incorporating advanced vascular imaging modalities, such as CT, MRI, and PET, to better characterise plaque burden and inflammation [31]. Additionally, inclusion of molecular and genetic biomarkers related to thrombosis and inflammation may help explain residual variance and improve risk stratification in diabetic CAD [32].

## Conclusions

The positive relationship between HbA1c and MPV observed in this cohort indicates that chronic hyperglycaemia may influence platelet size and reactivity, potentially through increased oxidative stress, reduced nitric-oxide bioavailability, and altered megakaryocyte maturation. The borderline indirect effect of HbA1C on coronary severity through MPV strengthens the concept that platelet morphology partly mediates the vascular consequences of poor glycaemic control. In contrast, the IPF did not correlate with either HbA1c or angiographic severity, suggesting that increased platelet turnover alone does not account for the observed pro-thrombotic state. These findings emphasise that MPV, rather than IPF, reflects the interplay between glycaemic dysregulation and platelet-driven atherothrombosis in diabetic CAD.

Longitudinal analysis of platelet and inflammatory marker changes over time would offer further insight into their role in the development and progression of CAD. Although this study contributes significantly to the existing body of literature, several limitations remain that need to be addressed by future research. One of them is the cross-sectional design of the study, which prohibits causality from being concluded, and the exclusive use of haematological markers will not reflect the full complexity of CAD pathology. The study lacked control over medication, lifestyle, and comorbidities affecting hematologic and inflammatory profiles. Future research should incorporate a broader range of clinical and genetic variables in CAD predictive models to enhance risk stratification. The findings emphasise the importance of considering multiple biological pathways in CAD severity assessment. While hematologic markers indicate inflammation, their direct role in assessing disease severity remains unclear. Future studies integrating genetic, metabolic, and imaging data may improve insights into CAD progression and cardiovascular risk prediction.

## Appendices

Appendix A 

**Table 1:** Descriptive statistics of key variables. MPV: mean platelet volume; IPF: immature platelet fraction; CAD: coronary artery disease; SVD: single vessel disease; DVD: double vessel disease; TVD: triple vessel disease

Variable	Mean ± SD	Min-Max
MPV (fL)	10.4 ± 1.5	7.8-13.1
IPF (%)	4.44 ± 0.46	3.1-5.5
Platelet Count (10^9^/L)	266 ± 71	154-404
Inflammatory marker (pg/mL)	2284 ± 656	1200-3700
Cholesterol (mg/dL)	169 ± 42	94-320
CAD severity	-	SVD: 47, DVD: 38, TVD: 45

Appendix B 

**Table 2:** Correlation matrix of key predictors. MPV: mean platelet volume

Variable	MPV	Cholesterol	Inflammatory Marker	Platelet Count
MPV	1.000	0.11	0.06	-0.29
Cholesterol	0.11	1.000	-0.04	-0.006
Inflammatory marker	0.06	-0.04	1.000	0.33
Platelet count	-0.29	-0.006	0.33	1.000

Appendix C 

**Table 3:** Structural Equation Model results. CAD: coronary artery disease; IPF: immature platelet fraction

Predictor	Estimate	Std. Error	z-value	P-value	Std. Estimate
CAD severity					
IPF	0.19	0.14	1.35	0.18	0.12
Inflammatory marker	0.16	0.33	0.49	0.62	0.04
Platelet count	0.05	0.25	0.21	0.83	0.02
Inflammatory marker					
IPF	0.09	0.03	2.55	0.011	0.20
Platelet count	0.27	0.06	4.35	<0.001	0.35
